# Design of Targeted Nanostructured Coordination Polymers (NCPs) for Cancer Therapy

**DOI:** 10.3390/molecules25153449

**Published:** 2020-07-29

**Authors:** Fernando Novio

**Affiliations:** Departament de Química, Universitat Autónoma de Barcelona, Campus UAB, Cerdanyola del Vallès, 08193 Barcelona, Spain; fernando.novio@uab.cat

**Keywords:** coordination polymers, nanomedicine, cancer therapy, drug delivery, EPR (Enhanced Permeability and Retention effect), tumor-targeting

## Abstract

Conventional cancer chemotherapy presents notable drug side effects due to non-selective action of the chemotherapeutics to normal cells. Nanoparticles decorated with receptor-specific ligands on the surface have shown an important role in improving site-selective binding, retention, and drug delivery to the cancer cells. This review summarizes the recent reported achievements using nanostructured coordination polymers (NCPs) with active targeting properties for cancer treatment in vitro and in vivo. Despite the controversy surrounding the effectivity of active targeting nanoparticles, several studies suggest that active targeting nanoparticles notably increase the selectivity and the cytotoxic effect in tumoral cells over the conventional anticancer drugs and non-targeted nanoparticle platform, which enhances drug efficacy and safety. In most cases, the nanocarriers have been endowed with remarkable capabilities such as stimuli-responsive properties, targeting abilities, or the possibility to be monitored by imaging techniques. Unfortunately, the lack of preclinical studies impedes the evaluation of these unique and promising findings for the translation of NCPs into clinical trials.

## 1. Introduction

Nanostructured Coordination Polymers (NCPs) represent a versatile class of nanomaterials based on the coordination of structural and functional organic ligands with metal ion nodes. The rich chemistry and infinite combination of different binomial metal-ligand units make these hybrid materials a fascinating class of nanomaterials with interesting applications. The self-assembly of NCPs with well-defined compositions and homogeneous sizes is a challenging area of growing interest. The chemical flexibility of these materials allows for systematically controlling and tuning their physicochemical properties and endowing them with interesting optical, magnetic, electronic, and catalytic properties [1,2]. The nanostructure of the coordination polymers has represented an opportunity to develop a unique class of highly tailorable functional materials. The nanometer size improves colloidal dispersion and increases the surface area, which allows a fine-tuning of the materials’ physicochemical properties [3].

Traditionally, the term NCPs has been used for amorphous nanoparticles and the crystalline and porous counterpart have been named as nanostructured metal-organic frameworks (NMOFs) [4]. Even though the crystalline nature of NMOFs facilitates the analysis of drug encapsulation, loading capacity and release studies are crucial for applications such as nanomedicine [5,6], NCPs represents an interesting alternative to NMOFs since, in recent years, several simple and reproducible synthetic methods for obtaining nano-constructs with high encapsulation capabilities as well as excellent colloidal and chemical stabilities were developed. Most of the NCPs were synthesized using fast precipitation methods by mixing metal ions and organic ligands in the presence of a poor solvent [7]. Since the seminal research works of Mirkin [8] and Wang [9] published in 2005, the number of publications related to NCPs have grown exponentially. This growth shows different applications of these versatile materials such as a heterogeneous catalyst [10,11,12], spin-crossover devices [13,14], (bio)sensors [15,16,17], or gas absorption systems [18,19] among others.

The special features and capabilities of NCPs have positioned them to be used in medical applications. These nanomaterials have special ability to integrate by physical encapsulation or chemical entrapment of a wide range of active species [20]. As a general trend, NCPs have shown controlled release properties of active principles such as organic dyes, drugs, or inorganic nanoparticles [21]. The principal advantage of these nano-systems lies in their chemical flexibility, which allows designing ligands susceptible to be cleaved under physiological conditions or in response of a specific external stimulus [22].

## 2. NCPs for Cancer Treatment 

The use of nanoparticles in cancer treatment has reduced the systemic toxicities and improved the pharmacokinetics of anticancer drugs through passive or selective targeting to tumors with the subsequent local delivery of the therapeutic agents. The fine tune of nanoparticles sizes, surface passivation, and surface coating with specific targeting molecules for the detection of tumoral cells have increased the anticancer action of well know anticancer drugs and the design of new nanodrugs. The ability of most of the nanoparticles to passively target tumors taking advantage of the leaky vasculatures in the tumoral area through the enhanced permeability and retention (EPR) effect, or the interaction of targeting molecules with specific receptors in the membrane of tumor cells (active targeting) has allowed increasing the therapeutic effect of the drugs by minimizing systemic toxicity or other side effects. Moreover, the ability of some nanoparticles to include different drugs in the same nano-construct with the possibility to modulate the release profiles can induce or maximize drugs′ synergistic effect.

An extensive number of publications and review articles have shown the progress in improving drug delivery pharmacokinetics and altering the drugs′ biodistribution by preferentially accumulating the nanoparticles in diseased tissues. The principal efforts have been focused on finding new strategies for incorporating different drugs into the coordination polymer matrix by controlling the drug release profiles and providing multifunctionality to the nano-formulation. The potential multifunctionality of these nano-systems and their chemical flexibility open new possibilities for their use as smart drug delivery systems, bioimaging probes, or a combination of both (theranostic applications). The increasing number of in vitro and in vivo studies concerning the use of NCPs for therapy and diagnosis in cancer treatment helps to understand their mechanism of action and their interaction with biological environments. As a result of these studies, a series of limitations and challenges have been highlighted as critical points for the clinical translation. 

**Figure 1:**
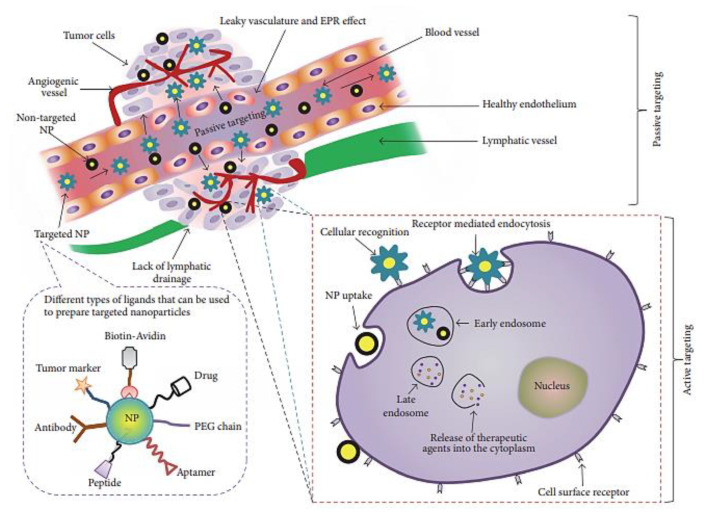
Schematic representation of passive and active targeting approaches. The figure is reproduced from Reference [27] with the permission from Hindawi.

Like many nanoparticles used in nanomedicine for cancer treatment, NCPs have the advantage of targeting cancer by simply being accumulated and entrapped in tumors by the EPR effect (passive targeting), which was caused by leaky angiogenetic vessels and poor lymphatic drainage [23]. Some studies have determined that the EPR effect is highly heterogeneous, which changes over time during tumor development and varies between mouse models and patients among tumor types [24]. Therefore, the treatment effectivity cannot be anticipated on the basis of preclinical studies. However, even if the overall median accumulation on nanoparticles in tumors is around a 0.7% injected dose, it seems sufficient and normally much higher than standard cytostatic compounds [25]. The use of Polyethyleneglycol (PEG) for coating the nanoparticles will protect the nanoparticles from the Reticulo-Endothelial System (RES). However, it seems to prevent cell uptake and the required intracellular drug release [26]. Additionally, the grafting of bio-recognition molecules onto the nanoparticles can induce the active targeting and increase specific cell uptake (Figure 1). Recognition of these ligands by cell surface receptors leads to receptor-mediated endocytosis. Several materials have been suggested for this purpose such as different polymers, folic acid, antibodies, aptamers, peptides, antibodies, or polysaccharides [27]. These ligands have been attached to the NCPs′ surface in a covalent or non-covalent manner and used with or without a targeting molecule.

There are several examples of successful cancer diagnostic and therapeutic nanoparticles, and many of them have rapidly moved to clinical trials [28]. Therefore, there is still room for optimization for NCPs in the area of the nanoparticle stability during plasma circulation, tumor bioavailability, and the study of the targeting ligands’ effect on their efficiency to treat cancer. NCPs acting via both mechanisms (EPR, active targeting) have been shown to increase drug concentration in cancer cells. However, active targeting nanoparticles have shown to be more efficient in increasing drug accumulation in cancer cells and cellular uptake playing an important role in cancer chemotherapy. The objective of this current review is to summarize the most relevant examples that demonstrated an efficient nano-construct for cancer treatment, especially in vivo.

## 3. Design of NCPs Nano-Formulations 

**Figure 2:**
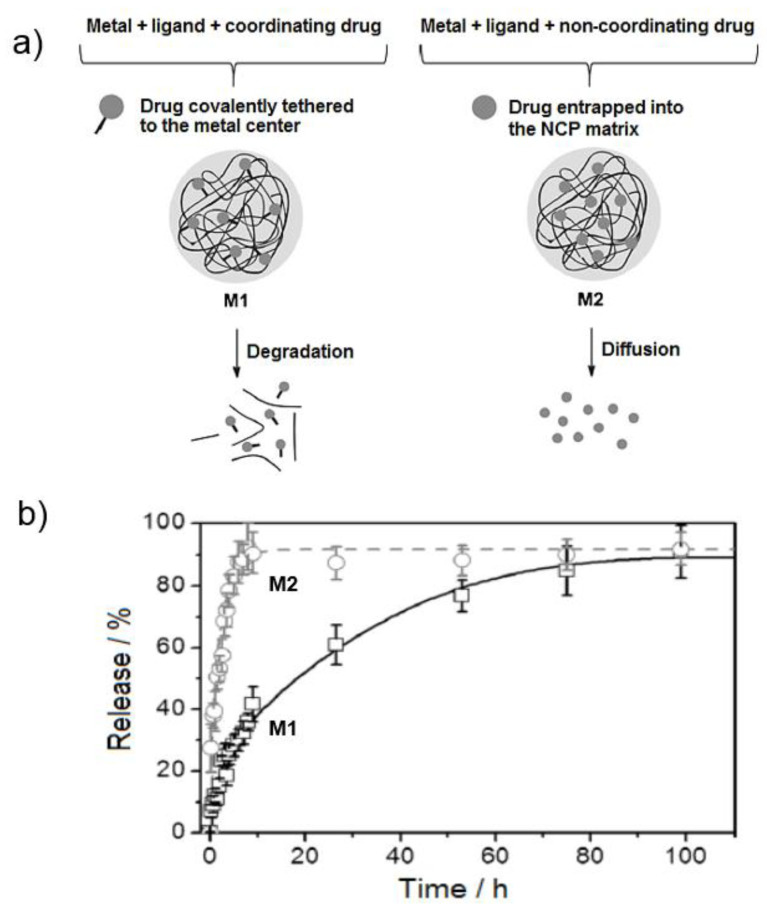
(**a**) Schematic of the nanostructured coordination polymers (NCPs) preparation containing a drug as a building block (M1) or entrapped into the polymeric matrix (M2). (**b**) Drug release profiles in aqueous media of guest molecules controlled by NCPs’ degradation (M1) or fast diffusion through the polymeric matrix (M2). The figure is reproduced from Reference [20] with the permission from John Wiley and Sons.

From 2005, when the first publications concerning the use of NCPs for biomedical applications were described, numerous articles were published showing the incorporation of multiple functionalities through the rational selection of specific metal ions and organic linkers. Various NCPs demonstrated their ability to co-encapsulate and deliver drugs and/or imaging agents in a controlled manner, which is difficult to achieve with other nano-formulations. Even though NCPs are not hollow or open-framework structures, their tunable matrix composition facilitates encapsulation of different functional species. The encapsulation usually proceeds through two distinct strategies: (1) using the drug as a building block integrated into the NCPs′ polymeric framework [29,30,31,32] and (2) physically entrapping the therapeutic agent within the polymeric matrix [33,34,35]. Accordingly, the release mechanism and release profile will be dictated by the particle degradation, fast diffusion processes, and/or a combination of both (Figure 2) [20].

Coordination bonding has emerged as a versatile method to prepare nano-complexes by assembling drugs into polymeric nanoparticles. Most of the studies showed that different metals can form coordination bonds with drugs such as daunorubicin or doxorubicin (DOX) [36], methotrexate (MTX) [37], bisphosphonates (N-BPs) [38,39], nicotinic acid [40], gossypol, and AQ4N [41] due to the presence of carboxyl, hydroxyl, aldehyde, or amino groups. Moreover, platinum-based chemotherapeutics (Cisplatin, carboplatin, and oxaliplatin) have often been used as building blocks in NCPs, and the combination of these platinum-based drugs with other chemotherapeutics has allowed to increase the synergistic therapy for treating multiple types of cancers including lung, breast, neuroblastoma, colorectal, pancreatic, cervical, and ovarian cancers, among others [42,43,44]. Additionally, in the last decade, an extensive study concerning the use of active metals such as ruthenium [45], copper [32,46], zinc, cobalt [47,48], or indium-based compounds [49] has received intensive attention as an alternative to cisplatin in order to reduce its cytotoxic effect and increase anticancer properties. The formed coordination bonds have adequate pH-response ability and can release drug molecules under small pH variations [50,51,52,53,54].

Many of the current nanomedicines, used for solid tumor treatment, accumulate in tumors due to the EPR effect. A thorough knowledge of the physical properties (e.g., size, surface charge, shape, and mechanical strength) and chemical attributes of nanoparticles facilitates their application in biomedicine [55]. Specifically, nanoparticles in the range 10–100 nm can passively target tumors by freely passing through large pores (40 nm to 1 um in size) and achieve higher intra-tumoral accumulation [56] due to the highly permeable blood vessels provoked by the rapid and defected angiogenesis and dysfunctional lymphatic drainage [57]. In this sense, although the wide variety of NCPs-based nano-systems are expected to accumulate in tumors through the EPR effect, one of the most challenging goals has been addressing these nano-constructs toward individualized therapy and cancer treatment, especially based on the identification of cancer biomarkers [58]. The design of strategies for detecting in vivo markers’ expression and monitoring them during treatment is a real challenge. In this scenario, nanoparticles decorated with specific targeting molecules can be designed for detecting extracellular cancer biomarkers, imaging tumors, and monitoring the treatment evolution. The objective is to obtain efficient nanoparticles that enormously help the pre-surgery and post-surgery cancer treatment.

In this review, we will summarize the most recent strategies for coating and surface functionalization of NCPs designed for passive and active targeting in cancer treatment.

### 3.1. Platinum-Based NCPs

Since resistance to the platinum chemotherapeutic agents is a major limitation for the successful treatment of many cancers, development of novel strategies to overcome intrinsic and acquired resistance to chemotherapy is of critical importance for the effective treatment of different types of cancers. In the case of NCPs, the exploration of non-classical mononuclear or polynuclear platinum(II) compounds, platinum(IV) prodrugs, or photoactivatable platinum (IV) complexes has afforded an intense number of research works including supramolecular self-assembled nanostructures, metal-organic frameworks, and diverse coordination polymer-based nano-formulations for developing the next generation of platinum cancer drugs.

Since 2008, Lin and coworkers have been developing a series of platinum-based NCPs nano-constructs to deliver platinum-based chemotherapeutics. Although the first publications were related to the synthesis, physicochemical characterization and study of NCPs’ cytotoxic effects [29,59,60]. In the last decade, the proposed nano-systems have increased in complexity, multifunctionality, selectivity, and effectivity at in vitro and in vivo levels. Most proposed nano-systems need a protective coating to avoid premature degradation and to increase colloidal stability in a physiological medium. One of the first examples of cancer targeting nanoparticles studied by Lin′s group detailed the synthesis of NCPs obtained from the coordination of Tb(III) ions with the cisplatin derivative c,c,t–(diamminedichlorodisuccinato)Pt (IV) [26]. The as-obtained nanoparticles were coated with a silica layer to stabilize the nanoparticles and increase the control on the release profile of the platinum (IV) prodrug. The silica coating was subsequently decorated with a silyl-derived peptide RGD and a small cyclic peptide with high binding affinity for α_ν_β_3_ integrin, which provided actively targeted cancer in vitro in the overexpressed HT-29 cell line. Although Pt (IV) prodrug remains inactive in physiological conditions, once inside the cells, the Pt (IV) complexes can become the pharmacologically active Pt (II) species by the action of reducing agents (i.e., ascorbic acid or glutathione) present in the intracellular environment [61]. The results suggested that the targeted nanoparticles were internalized much more than the non-functionalized ones presumably via receptor-mediated endocytosis.

**Figure 3:**
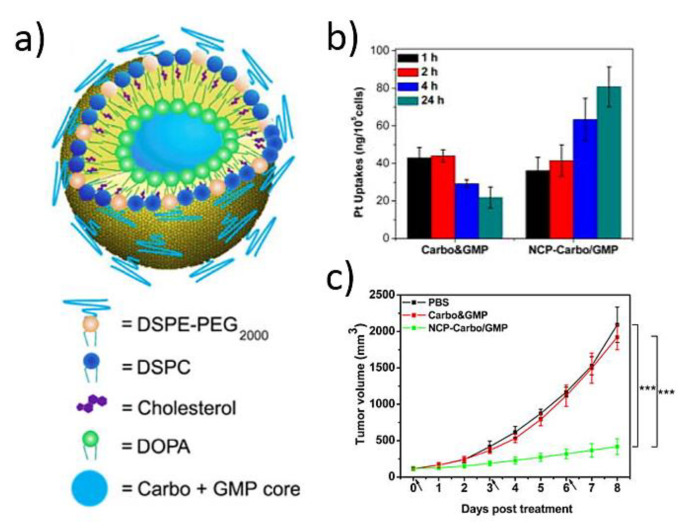
(**a**) Schematic of carboplatin and gemcitabine bearing NCPs (NCP-Carbo/GMP). (**b**) Cellular uptake of Carbo and GMP and NCP-Carbo/GMP in SKOV-3 cells determined by inductively coupled plasma mass spectrometry (ICP-MS). (**c**) In vivo tumor growth inhibition with a mixture Carbo and gemcitabine and NCP-Carbo/GMP administered on days 0, 3, and 6. Data are expressed as means ± SD (*n* = 5). The figure is reproduced from Reference [44] with the permission from American Chemical Society.

Apart from the silica coating, the stabilization with lipid coatings is one of the most common methodologies for conferring physical and chemical stability to NCPs. The lipid coating allows functionalizing the surface by targeting molecules such as anisamide (AA) to enhance cellular uptake and cytotoxicity in human cell lung cancer cells [62], or using a lipid coating intercalating PEG chains to minimize the nanoparticles’ clearance by the mononuclear phagocyte system and increase the blood circulation half-lives [63]. The studies with this kind of nanoparticles bearing different combinations of Pt (IV) prodrugs demonstrated the superior efficacy in treating different subcutaneous tumor murine models when compared with free drugs. This strategy was developed successfully to encapsulate and deliver in a control manner multiple chemotherapeutics for synergistic combination therapy including Pt (IV) prodrugs and gemcitabine in treating pancreatic or ovarian cancer [43,44] (Figure 3). The nanostructure of different drugs allows prolonged blood circulation half-life, improves tumor uptake, reduces the growth inhibition, and induces a practical regression of tumor cells.

Other nano-constructs have been designed for the co-delivering of chemotherapeutics and siRNAs to eradicate tumors of cisplatin-resistant cancer by overcoming intrinsic and acquired resistance to chemotherapy [64,65]. The NCP/siRNA combinations promote cellular uptake of cisplatin-derivatives and siRNA enable efficient endosomal escape in cisplatin-resistant ovarian cancer cells by downregulating the expression of multi-drug resistance genes. It has been demonstrated that the local administration of these nano-systems reduced tumor sizes of cisplatin-resistant subcutaneous xenografts. The combination of synergistic drugs using NCPs with targeting properties has received high attention since this strategy was presented as a solution for poor pharmacokinetics/dynamics, the rapid development of drug resistance, or the suppression of metastases [41,66,67].

Some examples of NCPs designed to target specific organs were published recently. In these systems, the use of precise biomolecules for improving the nanoparticles accumulation in the site of action has allowed the efficient delivery of therapeutics. Zhao and coworkers designed a cisplatin prodrug bearing Zn-based NCPs, which were coated with polyethylene glycol (PEG) and decorated with alendronate (ALN), which is a bone-seeking moiety [68]. The resulting nanoformulation showed much higher affinity for hydroxyapatite in vitro and bone in vivo than non-targeted nanoparticles. The in vivo biodistribution study after intravenous administration corroborated the increasing delivering (about four-fold) of platinum drug to the bone metastatic lesions in comparison with healthy bones. The treatment with these NCPs showed reduced toxicity of platinum, the inhibition of tumor growth, and the reduction of the osteo-calastic bone destruction.

Cancer immunotherapy is an interesting field in which the nanotechnology can afford different solutions [69]. It has been demonstrated that, in specific cases, nanoparticles can potentially stimulate tumor microenvironments to produce antitumor immunity. A very recent example was reported by Lin and coworkers who synthesized NCPs containing an immunostimulatory chemotherapeutic combination of Oxaliplatin (OxPt) and dihydroartemesinin (DHA). The NCP combination provoked an effective immunotherapy of colorectal cancer based on systemic drug delivery [70]. The authors developed a self-assembled NCPs carrying a Zn-OxPt coordination polymer coated by a lipid bilayer, which contained a cholesterol-DHA conjugate (chol-DHA). The chemotherapeutic activity combines the synergy of immune system activation by DHA [71,72,73], and the anticancer activity of Oxaliplatin. The favorable biodistribution, low cytotoxicity, and notable tumor uptake of these biodegradable NCPs allowed total tumor eradication in vivo. 

### 3.2. NCPs Including Non-Metal Chemotherapeutics

Apart from the ability of NCPs to encapsulate or integrate metal-based anticancer drugs (especially Pt-based drugs) as building blocks, these nanoparticles have demonstrated their capacity for encapsulating small chemotherapeutics by physical entrapment into the NCPs′ matrix or using the coordination ability of some organic drugs to be included as bridging ligands in the coordination polymer network. As previously mentioned, the mode of drug incorporation will determine the release profile of the nano-construct [20]. Usually, the encapsulation yield through physical entrapment is lower (20% in weight) in comparison with the chemical inclusion (up to 80%). Most of the encapsulated chemotherapeutics using NCPs include drugs that induce cell apoptosis such as Methotrexate (MTX) [37], prevent DNA replication such as doxorubicin (DOX) [74,75] or others used in treating multiple cancer types (5-Fluorouracil [76], bisphosphonates [39,77], and camptothecin [34]).

The lability of coordination bonds in NCPs facilitates the fabrication of nano-systems with responsive properties that may be used to improve the stability in physiological environments and control of targeted delivery. This pH-response behavior is of special relevance due to the pH changes in cancer cells and tumoral environments. Taking advantage of these properties, a vast number of research works have been developed to assure a rapid and quantitative release in tumors subject to pH regulation [36,37].

**Figure 4:**
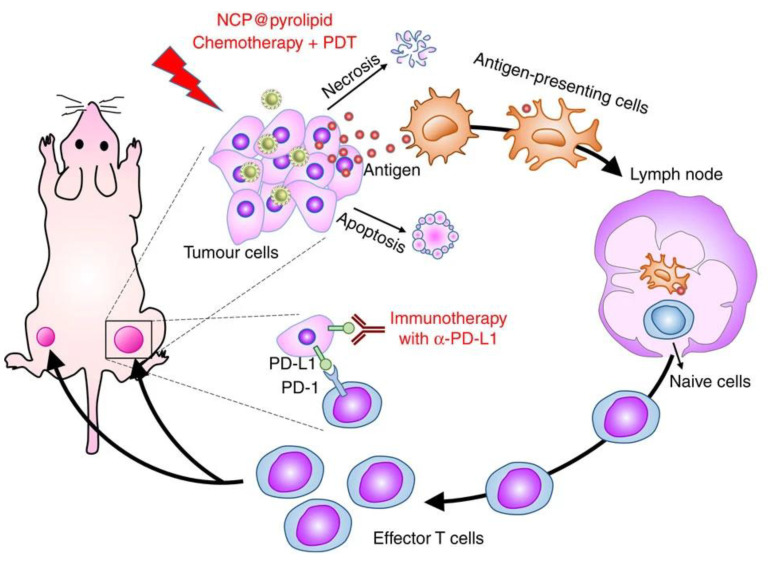
Schematic of the combined action (Chemotherapy + PDT) of nanostructured coordination polymers (NCP) @pyrolipid able to induce immunogenic cell death and an inflammatory environment at the primary tumor site, which leads to the release of tumor-associated antigens (TAAs). The result of this action is not only tumor eradication in the primary sites but also a systemic antitumor immune response to reject distant tumors. The figure is reproduced from Reference [82] with the permission from Springer Nature.

Despite the therapeutic importance and targeting properties of drug-contained NCPs in vitro [78,79,80], few examples reported a study of NCPs concerning their in vivo antitumoral activity or its use as targeted multidrug delivery systems for cancer treatment. However, there is an increasing interest in designing multifunctional nano-formulations including a mixture of synergic chemo-therapeuticals and active multitherapy effect to improve anticancer efficacy. Combination therapy is proposed as an alternative to reduce drug resistance and increasing the therapeutic effect obtained by using the drugs separately. An interesting example is found in the nano-system proposed by Lin and coworkers who reported a core–shell NCPs obtained by the coordination of Zn (II) ions with a Pt (IV) cisplatin prodrug coated by the first lipid layer. These primary nanoparticles were coated by an outer lipid layer containing PEG chains and pyro-lipid photosensitizer to use them in combined chemotherapy and photodynamic therapy (PDT) [81]. The controlled release of cisplatin and pyrolipid molecules induce cancer cell apoptosis and necrosis in vivo, and high tumor regression efficacy in a subcutaneous xenograft mouse model of resistant head and neck cancer in comparison to the administration of the drugs separately. Although the nanoparticles do not have a targeting molecule on the surface, the PEG chains induce a prolonged systemic distribution and tumor accumulation by taking advantage of the EPR effect. This study suggests multifunctional NCP core-shell nanoparticles act as a versatile and effective drug delivery system for incorporating multiple therapeutic agents or modalities that treat many complex cancers. The same kind of nanoparticles are able to induce a strong tumor-specific immune response in advanced colorectal cancer models in vivo, which demonstrated their enormous multifunctionality and effectivity (Figure 4) [82].

Another example of NCPs effective in the combination of chemotherapy and PDT was reported by Liu and coworkers recently. The authors presented a type of NCPs formed by the coordination of Cu (II) ions with the banoxantrone (AQ4N) and doxorubicin [83]. The inherent porosity of the resulting material is able to encapsulate a photosensitize (phthalocyanine, ZnPC). In this case, the chemotherapeutic efficacy is induced by “on-demand” photodynamic therapy since the photosensitization of ZnPC is quenched by Cu (II) until the acidic-based cleavage of coordination bonds in the tumor environment and ZnPC recover the photo-properties. Afterward, the PDT effect further induces a hypoxic environment necessary to enhance the reduction of AQ4N and increase the therapeutic efficiency. This is a clear example of a programmable nano-formulation able to control the synergistic effect of the therapeutic agents for cancer therapy. Although the nanoparticles were no covered with a specific coating or targeting molecules, the biodistribution analysis in vivo in HepG2 tumor-bearing mice showed a prolonged blood circulation and distribution in different organs with nonspecific preference for the tumor. However, a deeper and better penetration in tumors were observed in comparison with the free drugs administered separately.

### 3.3. Development of Targeting NCPs for Photodynamic Therapy

Although PDT is proposed in combination with chemotherapy, various NCPs designs have been developed to deliver PDT agents to tumors. However, only a few of them achieved a clinically relevant anticancer efficacy. Apart from the photosensitizer-containing porous metal–organic framework (MOF) nano-systems [84,85], some unique examples of NCPs have also been found in recent publications.

PDT is an effective anticancer procedure that involves the administration of a tumor-localizing photosensitizer (PS) that can be activated by light activation to generate highly cytotoxic reactive oxygen species (ROS) in situ. Usually the ROS production is related to generating singlet oxygen (^1^O_2_), which is a specie able to provoke cell apoptosis/necrosis and induce antitumor immunity [86]. The development of PS-containing NCPs with specific tumor-targeting abilities are interesting candidates to selectively release the photoactive molecules in the tumor cells preserving adjacent tissues. Although some of the limitations of this therapy is related to the difficulty of using a local light irradiation source in deep tumors, it cannot cure advanced disseminated disease because irradiation of the whole body with appropriate doses is not currently possible. However, for early or localized tumors, PDT can be a selective and curative therapy with many potential advantages over available alternatives.

Recent studies have afforded substantial advances in understanding the behavior of light in human tissues [87,88] and in the development of equipment for light delivery of PDT [89]. The last advancement has allowed designing methodologies to apply the optimal light irradiation (wavelength and intensity) in internal and external tumors using endoscopy or interstitial treatment to channeling light into optical fiber. PDT is now rarely rejected because of difficulties in light delivery. The key parameters that determine PDT efficacy are the nature of the photosensitizer, the intensity and wavelength of the light, and the tumor microenvironment. Over the years, several groups aimed to monitor and manipulate the components of these critical parameters to improve the effectiveness of PDT treatments. Thus, most of the NCPs’ designs are developed to take into account these parameters to try to improve the clinical utility of PDT as a powerful modality for treating cancer.

**Figure 5:**
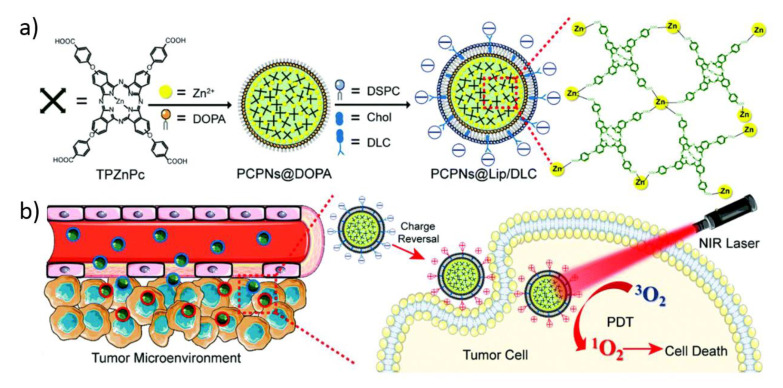
(**a**) Schematic representation of the synthesis and application of photodynamic therapy (PDT) active nanostructured coordination polymers (NCPs). (**a**) Preparation of the nanoparticles, and (**b**) accumulation in the tumor after intravenous administration, which is followed by its charge-reversal ability to improve the selective uptake and effective PDT in tumor cells after laser irradiation. The figure is reproduced from Reference [90] with the permission from American Chemical Society.

One of the most interesting examples have been published by Zhao and coworkers. The authors designed NCPs containing tetra(4-carboxyphenoxy)-phthalocyaninatozinc(II) (TPZnPc) photosensitizer coordinated with zinc ions. The preformed nanoparticles were coated with a lipid bilayer containing 1,2-dicarboxylic-cyclohexane anhydride modified lysyl-cholesterol (DLC) that promotes tumor cellular uptake through an electrostatic interaction induced by the mildly acidic tumor microenvironment [90]. These NCPs were proved to enhance selective tumor cellular uptake and generate more intracellular ^1^O_2_ after irradiation in vivo, which demonstrates the potential application of these NCPs in effective PDT (Figure 5).

A more complex and multifunctional nano-construct has been developed by Chen and coworkers using DNA-based NCPs obtained by the coordination of Ca (II) ions and AS1411 DNA G quadruplexes functionalized with chlorine e6 (Ce6) photosensitizer, hemin, and iron-containing porphyrin [91]. Further polyethylene glycol (PEG) functionalization enabled NCPs for improving the intranuclear transport of photosensitizer Ce6 and generating ROS inside tumor cells. Additionally, the inhibition of antiapoptotic protein B-cell lymphoma 2 (Bcl-2) expression by AS1411 allowed for greatly improving PDT-induced cell apoptosis. The catalase-mimicking DNAzyme function of G-quadruplexes and hemin could decompose tumor endogenous H_2_O_2_ to in situ generated oxygen in order to further enhance PDT by overcoming the hypoxia-associated resistance. This design showed an interesting concept of multifunctional NCPs for combining drug delivery properties with a PDT effect, down-regulation of antiapoptotic proteins, and the modulation of the unfavorable tumor microenvironment simultaneously.

### 3.4. Other Explored Applications of NCPs in Cancer Treatment

The use of NCPs has opened new avenues in the design of new therapeutic tools and strategies to treat many cancers. Different new approximations try to solve problems related to the physiology of tumor extracellular matrix (ECM) to improve the tumor retention and penetration of therapeutic agents [92], the use of NCPs as biocompatible gene-regulation agents [93], obtaining of active nanoparticles in radioisotope therapy (RIT) for clinical cancer treatment [94], or the encapsulation of individual tumor cells in biocompatible coordination polymers, as whole cell vaccines, to regulate the immune system and combat metastatic cancer [95]. All these very recent examples prelude an intense and prolific research field that make us think that the clinical use of these nano-systems is likely not so far.

The development of hybrid nano-systems combining coordination polymers with other materials has increased the pool of materials with biomedical applications using novel approximations with high potentiality. Thus, we can found some examples like the coordination polymer derived from (pq)_2_Ir(Hdcbpy) and Dy(OOCCH_3_)_3_ hybridized Au nanocages (AuNC@CPs) that offer excellent photothermal, photoacoustic, and magnetic properties in solution. These special features make AuNC@CPs interesting materials for near-infrared (NIR)-driven photothermal therapy (PTT) guided by photoacoustic (PA) and magnetic resonance (MR) imaging in vivo [96]. Other interesting examples include DNA-Iron based NCPs tailored for the delivery of functional DNA to cells in vitro and in vivo and used for diagnostic and therapeutic applications [97].

Some of the recent publications have shown interesting multifunctional nanoplatforms, such as that reported by Liu and coworkers, which detailed a biomimetic nanoprobe based on a pH-responsive porous NCPs for activated fluorescence imaging and targeted drug delivery [85]. The reported NCPs were synthesized by the coordination of polyphenol ligands and dopamine with Fe(III) ions. Subsequently, the NCPs were loaded with DOX through physical and electrostatic absorption, and then the nanoparticles were coated with a cell membrane extracted from Bel-7402 cancer cells, which have been demonstrated to afford the ability to evade phagocytosis and enhance specific endocytosis by homotypic cells [98]. The fluorescence of DOX was quenched due to the fluorescence resonance energy transfer between DOX and the coordination polymeric matrix. However, under an acidic environment inside cancer cells, NCPs were degraded, DOX was released, and the fluorescence of DOX was activated. The resulting NCPs significantly enhanced the cellular endocytosis of DOX in Bel-7402 cancer cells and exhibited an excellent cancer therapy effect in vitro, which demonstrated that this platform is useful for an activated cancer imaging and personalized cancer treatment.

Another interesting example of multifunctional and synergetic tumor-targeted nanoparticles were reported by Pan and coworkers. The authors reported a chemo-photothermal combined therapeutic nanoplatform based on PEGylated borate-coordination-polymer coated with polydopamine (PDA@CP-PEG) [99]. The nanoparticles exhibited a synergetic targeting property for the sialic acid-overexpressed tumor cells since PEG improve the passive targeting and phenylboronic acid (PBA) molecules induce the active targeting due to the weak-acid stable interaction of PBA and sialic acid under the acidic tumor microenvironment. In addition, the photothermal effect of the polydopamine core and DOX drug release properties endow the nanoparticles with the potential for chemo-photothermal combination therapy. In vitro and in vivo studies demonstrated the multifunctional properties of these NCPs with efficient tumor targeting, low toxicity, and excellent chemo-photothermal activity for tumor inhibition. These types of multifunctional nanoparticles provided an insight into the development of a high-efficiency anti-tumor nanoplatform for potential clinical applications.

## 4. Conclusions and Future Perspective

As observed previously, there are few examples of active targeting for cancer treatment in the case of NCPs. However, currently, there are many types of nanoparticles at an early design step that may progress in the future to preclinical development for cancer imaging and therapy. A particular version of these nanoparticles is the targeting-active nanoparticles. In this scenario, there is still plenty of room for improvement.

The local delivery of a specific drug, or a combination of synergic drugs, can lead to cell killing in a very specific tumor in a short period of time. The development of more effective and selective therapies would avoid surgery and/or chronic chemotherapy treatments by increasing the quality and life expectancy of the patient. Thus, more efforts should be put in developing new types of NCPs’ coatings (i.e., lipids, polymers, and inorganic coatings) to increase the physicochemical stability of the nanoparticles in physiological environments, and design new manners to decorate the nanoparticles’ surface with specific targeting molecules (i.e., hyaluronic acid, folic acid, antibodies, aptamers, carbohydrates, or polysaccharides) to adequately treat specific cancer types. There are important factors that should be revised and studied such as the performance of PEG-coated nanoparticles (PEG size and its grafting density) in order to design and apply for optimum circulation times and tumor cell uptake. In the same way, it is important to design new targeting ligands to avoid macrophage recognition and faster clearance compared to the non-targeted nanoparticles. The objective is to obtain smart nanoparticles bearing a cleavable masking of the ligands until reaching the tumor cells.

Complementarily, there is a need to study different administration routes (i.e., intravenous, oral, intranasal, etc.) as well as the mechanism of clearance, metabolism, and excretion of nanoparticles and their components. Active targeting NCPs containing chemotherapeutic drugs have been demonstrated in various studies, both in vitro and in vivo, to improve selectivity of cellular uptake through receptor-mediated endocytosis. This fact represents several advantages concerning drug efficacy (selective action to cancer cells) and safety (avoid of side effects) over the conventional chemotherapeutic drugs and non-targeted nanoparticles. However, the main limitation for the clinical application is that their use is limited to only certain types of cancer that express specific receptors on the cell surfaces. Selection of the targeting nanoparticles is determined by the types of target proteins or receptors expressed on cancer cell surfaces. Clinical studies are required to confirm their application in cancer patients.

NCPs for cancer therapy have evolved enormously during the last decade and it is expected to end up in clinical assays and clinical practice in a short time. Therefore, it is necessary for expert and emergent research groups in areas such as chemistry, biology, medicine, chemical engineering, among others, in order to optimize many of the proposed examples and new ones that may reach clinical application. The control of the physicochemical properties of NCPs and the exploitation of multifunctionality aspects are the main reasons for their increasing applications as anticancer agents. Moreover, there is still a lack of knowledge in the control of biodegradation and metabolic processes, the interaction with the immune system, and the excretion mechanisms. From a technical point of view, there are still some limitations concerning the cost of production and manufacturing of nanoparticle platforms that require sophisticated technology.

Since the early 1990s, nanoparticle drug delivery systems have been used in the clinic to improve the delivery of different therapeutics. Over past decades, newer generations of nanoparticles like NCPs have emerged to try to reach clinical trials and be approved for various applications. However, there is still a long way toward the complete regulation of nanomedicines. The need for more sophisticated nanostructured designs also requires a careful understanding of pharmacokinetic and pharmacodynamic properties of nanomedicines, determined by the respective chemical composition and physicochemical properties, which poses additional challenges in regulatory terms. To avoid any concern, it is necessary to establish an unambiguous definition of nanomaterials and work on the regulatory process to facilitate the reach of nanoparticles to the market and the clinical use.

## References

[1] Batten S.R., Neville S.M., Turner D.R. (2009). Coordination Polymers: Design, Analysis and Application.

[2] Mu J., He L., Huang P., Chen X. (2019). Engineering of nanoscale coordination polymers with biomolecules for advanced applications. Coord. Chem. Rev..

[3] Chen L.J., Yang H.B. (2018). Construction of Stimuli-Responsive Functional Materials via Hierarchical Self-Assembly Involving Coordination Interactions. Acc. Chem. Res..

[4] Giménez-Marqués M., Hidalgo T., Serre C., Horcajada P. (2016). Nanostructured metal–organic frameworks and their bio-related applications. Coord. Chem. Rev..

[5] Dang S., Zhu Q.L., Xu Q. (2017). Nanomaterials Derived from Metal-Organic Frameworks. Nat. Rev. Mater..

[6] Sun W., Li S., Tang G., Luo Y., Ma S., Sun S., Ren J., Gong Y., Xie C. (2019). Recent Progress of Nanoscale Metal-Organic Frameworks in Cancer Theranostics and the Challenges of Their Clinical Application. Int. J. Nanomed..

[7] Morsali A., Hashemi L. (2020). Chapter Two—Nanoscale coordination polymers: Preparation, function and application. Adv. Inorg. Chem..

[8] Oh M., Mirkin C.A. (2005). Chemically Tailorable Colloidal Particles from Infinite Coordination Polymers. Nature.

[9] Sun X., Dong S., Wang E. (2005). Coordination-Induced Formation of Submicrometer-Scale, Monodisperse, Spherical Colloids of Organic-Inorganic Hybrid Materials at Room Temperature. J. Am. Chem. Soc..

[10] Park K.H., Jang K., Son S.U., Sweigart D.A. (2006). Self-Supported Organometallic Rhodium Quinonoid Nanocatalysts for Stereoselective Polymerization of Phenylacetylene. J. Am. Chem. Soc..

[11] Mohammadikish M., Yarahmadi S. (2020). New self-supporting heterogeneous catalyst based on infinite coordination polymer nanoparticles. J. Phys. Chem. Solids.

[12] Li W., Li F., Yang H., Wu X., Zhang P., Shan Y., Sun L. (2019). A bio-inspired coordination polymer as outstanding water oxidation catalyst via second coordination sphere engineering. Nat. Commun..

[13] Puigmartí-Luis J. (2014). Microfluidic Platforms: A Mainstream Technology for the Preparation of Crystals. Chem. Soc. Rev..

[14] Salmon L., Catala L. (2018). Spin-crossover nanoparticles and nanocomposite materials. C. R. Chim..

[15] Huang P., Mao J., Yang L., Yu P., Mao L. (2011). Bioelectrochemically Active Infinite Coordination Polymer Nanoparticles: One-Pot Synthesis and Biosensing Property. Chem. Eur. J..

[16] Deng J., Wu F., Yu P., Mao L. (2018). On-site sensors based on infinite coordination polymer nanoparticles: Recent progress and future challenge. Appl. Mater. Today.

[17] Tan H., Liu B., Chen Y. (2012). Lanthanide Coordination Polymer Nanoparticles for Sensing of Mercury(II) by Photoinduced Electron Transfer. ACS Nano.

[18] Jeon Y.M., Armatas G.S., Heo J., Kanatzidis M.G., Mirkin C.A. (2008). Amorphous Infinite Coordination Polymer Microparticles: A New Class of Selective Hydrogen Storage Materials. Adv. Mater..

[19] Bennett T.D., Horike S. (2018). Liquid, glass and amorphous solid states of coordination polymers and metal–organic frameworks. Nat. Rev. Mater..

[20] Amorín-Ferré L., Busqué F., Bourdelande J.L., Ruiz-Molina D., Hernando J., Novio F. (2013). Encapsulation and Release Mechanisms in Coordination Polymer Nanoparticles. Chem. Eur. J..

[21] Novio F., Simmchen J., Vázquez-Mera N., Amorín-Ferré L., Ruiz-Molina D. (2013). Coordination Polymer Nanoparticles in Medicine. Coord. Chem. Rev..

[22] Nador F., Novio F., Ruiz-Molina D. (2014). Coordination Polymer Particles with Ligand-Centred PH-Responses and Spin Transition. Chem. Commun..

[23] Golombek S.K., May J.-N., Theek B., Appold L., Drude N., Kiessling F., Lammers T. (2018). Tumor Targeting via EPR: Strategies to Enhance Patient Responses. Adv. Drug Deliv. Rev..

[24] Tanaka N., Kanatani S., Tomer R., Sahlgren C., Kronqvist P., Kaczynska D., Louhivuori L., Kis L., Lindh C., Mitura P. (2017). Whole-tissue biopsy phenotyping of three-dimensional tumours reveals patterns of cancer heterogeneity. Nat. Biomed. Eng..

[25] Wilhelm S., Tavares A.J., Dai Q., Ohta S., Audet J., Dvorak H.F., Chan W.C.W. (2016). Analysis of nanoparticle delivery to tumours. Nat. Rev. Mater..

[26] Ahn S., Seo E., Kim K., Lee S.J. (2013). Controlled cellular uptake and drug efficacy of nanotherapeutics. Sci. Rep..

[27] Jahan S.T., Sadat S.M.A., Walliser M., Haddadi A. (2017). Targeted Therapeutic Nanoparticles: An Immense Promise to Fight against Cancer. J. Drug Deliv..

[28] Anselmo A.C., Mitragotri S. (2019). Nanoparticles in the clinic: An update. Bioeng. Transl. Med..

[29] Rieter W.J., Pott K.M., Taylor K.M.L., Lin W. (2008). Nanoscale Coordination Polymers for Platinum-Based Anticancer Drug Delivery. J. Am. Chem. Soc..

[30] Yang J., Liu W., Sui M., Tang J., Shen Y. (2011). Platinum (IV)-Coordinate Polymers as Intracellular Reduction-Responsive Backbone-Type Conjugates for Cancer Drug Delivery. Biomaterials.

[31] Solórzano R., Tort O., García-Pardo J., Escribà T., Lorenzo J., Arnedo M., Ruiz-Molina D., Alibés R., Busqué F., Novio F. (2019). Versatile Iron-Catechol-Based Nanoscale Coordination Polymers with Antiretroviral Ligand Functionalization and Their Use as Efficient Carriers in HIV/AIDS Therapy. Biomater. Sci..

[32] Wang K., Ma X., Shao D., Geng Z., Zhang Z., Wang Z. (2012). Coordination-Induced Assembly of Coordination Polymer Submicrospheres: Promising Antibacterial and in Vitro Anticancer Activities. Cryst. Growth Des..

[33] Imaz I., Rubio-Martínez M., García-Fernández L., García F., Ruiz-Molina D., Hernando J., Puntes V., Maspoch D. (2010). Coordination Polymer Particles as Potential Drug Delivery Systems. Chem. Commun..

[34] Novio F., Lorenzo J., Nador F., Wnuk K., Ruiz-Molina D. (2014). Carboxyl Group (-CO_2_H) Functionalized Coordination Polymer Nanoparticles as Efficient Platforms for Drug Delivery. Chem. Eur. J..

[35] Xing L., Zheng H., Cao Y., Che S. (2012). Coordination Polymer Coated Mesoporous Silica Nanoparticles for pH-Responsive Drug Release. Adv. Mater..

[36] Xing L., Zheng H., Che S. (2011). A pH-responsive cleavage route based on a meta-organic coordination bond. Chem. Eur. J..

[37] Xing L., Cao Y., Che S. (2012). Synthesis of core–shell coordination polymernanoparticles (CPNs) for pH-responsive controlled drug release. Chem. Commun..

[38] Green J.R. (2004). Bisphosphonates: Preclinical Review. Oncologist.

[39] Wang S., Della Rocca J., Lin W., Huxford-Phillips R.C., Kramer S.A., Liu D. (2012). Coercing Bisphosphonates to Kill Cancer Cells with Nanoscale Coordination Polymers. Chem. Commun..

[40] Etaiw S.E.H., Fayed T.A., El-bendary M.M., Marie H. (2018). Three-dimensional coordination polymers based on trimethyltin cation with nicotinic and isonicotinic acids as anticancer agents. Appl. Organomet. Chem..

[41] Shen S., Wu Y., Li K., Wang Y., Wu J., Zeng Y., Wu D. (2018). Versatile Hyaluronic Acid Modified AQ4N-Cu(II)-Gossypol Infinite Coordination Polymer Nanoparticles: Multiple Tumor Targeting, Highly Efficient Synergistic Chemotherapy, and Real-Time Self-Monitoring. Biomaterials.

[42] Adarsh N.N., Frias C., Ponnoth Lohidakshan T.M., Lorenzo J., Novio F., Garcia-Pardo J., Ruiz-Molina D. (2018). Pt(IV)-based nanoscale coordination polymers: Antitumor activity, cellular uptake and interactions with nuclear DNA. Chem. Eng. J..

[43] Poon C., He C., Liu D., Lu K., Lin W. (2015). Self-Assembled Nanoscale Coordination Polymers Carrying Oxaliplatin and Gemcitabine for Synergistic Combination Therapy of Pancreatic Cancer. J. Control. Release.

[44] Poon C., Duan X., Chan C., Han W., Lin W. (2016). Nanoscale Coordination Polymers Codeliver Carboplatin and Gemcitabine for Highly Effective Treatment of Platinum-Resistant Ovarian Cancer. Mol. Pharm..

[45] Li J., Murakami T., Higuchi M. (2013). Metallo-Supramolecular Polymers: Versatile DNA Binding and Their Cytotoxicity. J. Inorg. Organomet. Polym. Mater..

[46] Lakshmipraba J., Arunachalam S., Riyasdeen A., Dhivya R., Vignesh S., Akbarsha M.A., James R.A. (2013). DNA/RNA Binding and Anticancer/Antimicrobial Activities of Polymer-Copper(II) Complexes. Spectrochim. Acta Part A Mol. Biomol. Spectrosc..

[47] Nehru S., Arunachalam S., Arun R., Premkumar K. (2014). Polymer-Cobalt(III) Complexes: Structural Analysis of Metal Chelates on DNA Interaction and Comparative Cytotoxic Activity. J. Biomol. Struct. Dyn..

[48] Raja D.S., Bhuvanesh N.S.P., Natarajan K. (2012). Novel Water Soluble Ligand Bridged Cobalt(II) Coordination Polymer of 2-Oxo-1,2-Dihydroquinoline-3-Carbaldehyde (Isonicotinic) Hydrazone: Evaluation of the DNA Binding, Protein Interaction, Radical Scavenging and Anticancer Activity. Dalt. Trans..

[49] Shu D., Chen W. (2018). Synthesis, Structure, and in Vitro Anti-Lung Cancer Activity on an In-Based Nanoscale Coordination Polymer. Main Group Met. Chem..

[50] Bai L., Song F., Wang X.H., Cao J.Y.Q., Han X., Wang X.L., Wang Y.Z. (2015). Ligand-Metal-Drug Coordination Based Micelles for Efficient Intracellular Doxorubicin Delivery. RSC Adv..

[51] Han K., Zhang W.Y., Zhang J., Ma Z.Y., Han H.Y. (2017). pH-Responsive Nanoscale Coordination Polymer for Efficient Drug Delivery and Real-Time Release Monitoring. Adv. Healthc. Mater..

[52] Xu S., Liu J., Li D., Wang L., Guo J., Wang C., Chen C. (2014). Fe-Salphen Complexes from Intracellular pH-Triggered Degradation Of Fe_3_O_4_@Salphen-InIII CPPs for Selectively Killing Cancer Cells. Biomaterials.

[53] Wang T., Liu X., Zhu Y., Cui Z.D., Yang X.Z., Pan H., Yeung K.W.K., Wu S. (2017). Metal Ion Coordination Polymer-Capped pH-Triggered Drug Release System on Titania Nanotubes for Enhancing Self-Antibacterial Capability of Ti Implants. ACS Biomater. Sci. Eng..

[54] Ju Y., Cui J., Sun H., Müllner M., Dai Y., Guo J., Bertleff-Zieschang N., Suma T., Richardson J.J., Caruso F. (2016). Engineered Metal-Phenolic Capsules Show Tunable Targeted Delivery to Cancer Cells. Biomacromolecules.

[55] Zein R., Sharrouf W., Selting K. (2020). Physical properties of nanoparticles that result in improved cancer targeting. J. Oncol..

[56] Kang H., Rho S., Stiles W.R., Hu S., Baek Y., Hwang D.W., Kashiwagi S., Kim M.S., Choi H.S. (2020). Size-dependent EPR effect of polymeric nanoparticles on Tumor targeting. Adv. Healthc. Mater..

[57] Thanou M., Duncan R. (2003). Polymer-protein and polymer-drug conjugates in cancer therapy. Curr. Opin. Investig. Drugs.

[58] Evans W.E., Relling M.V. (2004). Moving towards individualized medicine with pharmacogenomics. Nature.

[59] Jhonstone T.C., Suntharalingam K., Lippard S.J. (2016). The next generation of platinum drugs: Targeted Pt(II) agents, nanoparticles delivary, and Pt(IV) prodrugs. Chem. Rev..

[60] Jeong Y.H., Shin H.W., Kwon J.Y., Lee S.M. (2018). Cisplatin-Encapsulated Polymeric Nanoparticles with Molecular Geometry-Regulated Colloidal Properties and Controlled Drug Release. ACS Appl. Mater. Interfaces.

[61] Avan A., Postma T.J., Ceresa C., Avan A., Cavaletti G., Giovannetti E., Peters G.J. (2015). Platinum-Induced Neurotoxicity and Preventive Strategies: Past, Present, and Future. Oncologist.

[62] Huxford-Phillips R.C., Russell S.R., Liu D., Lin W. (2013). Lipid-Coated Nanoscale Coordination Polymers for Targeted Cisplatin Delivery. RSC Adv..

[63] Liu D., Poon C., Lu K., He C., Lin W. (2014). Self-Assembled Nanoscale Coordination Polymers with Trigger Release Properties for Effective Anticancer Therapy. Nat. Commun..

[64] He C., Poon C., Chan C., Yamada S.D., Lin W. (2016). Nanoscale Coordination Polymers Codeliver Chemotherapeutics and siRNAs to Eradicate Tumors of Cisplatin-Resistant Ovarian Cancer. J. Am. Chem. Soc..

[65] He C., Liu D., Lin W. (2015). Self-Assembled Nanoscale Coordination Polymers Carrying SiRNAs and Cisplatin for Effective Treatment of Resistant Ovarian Cancer. Biomaterials.

[66] Hu Y., Lv T., Ma Y., Xu J., Zhang Y., Hou Y., Huang Z., Ding Y. (2019). Nanoscale Coordination Polymers for Synergistic NO and Chemodynamic Therapy of Liver Cancer. Nano Lett..

[67] Chan C., Guo N., Duan X., Han W., Xue L., Bryan D., Wightman S.C., Khodarev N.N., Weichselbaum R.R., Lin W. (2019). Systemic MiRNA Delivery by Nontoxic Nanoscale Coordination Polymers Limits Epithelial-to-Mesenchymal Transition and Suppresses Liver Metastases of Colorectal Cancer. Biomaterials.

[68] He Y., Huang Y., Huang Z., Jiang Y., Sun X., Shen Y., Chu W., Zhao C. (2017). Bisphosphonate-Functionalized Coordination Polymer Nanoparticles for the Treatment of Bone Metastatic Breast Cancer. J. Control Release.

[69] Velpurisiva P., Gad A., Piel B., Jadia R., Rai P. (2017). Nanoparticle Design Strategies for Effective Cancer Immunotherapy. J. Biomed..

[70] Duan X., Chan C., Han W., Guo N., Weichselbaum R.R., Lin W. (2019). Immunostimulatory Nanomedicines Synergize with Checkpoint Blockade Immunotherapy to Eradicate Colorectal Tumors. Nat. Commun..

[71] Zhang T., Zhang Y., Jiang N., Zhao X., Sang X., Yang N., Feng Y., Chen R., Chen Q. (2020). Dihydroartemisinin regulates the immune system by promotion of CD8+ T lymphocytes and suppression of B cell responses. Sci. China Life Sci..

[72] Kim S.H., Kang S.H., Kang B.S. (2016). Therapeutic effects of dihydroartemisinin and transferrin against glioblastoma. Nutr. Res. Pract..

[73] Yao W., Wang F., Wang H. (2016). Immunomodulation of artemisinin and its derivatives. Sci. Bull..

[74] Imaz I., Hernando J., Ruiz-Molina D., Maspoch D. (2009). Metal-Organic Spheres as Functional Systems for Guest Encapsulation. Angew. Chem. Int. Ed..

[75] Fan C., Wang D.A. (2016). Novel Gelatin-Based Nano-Gels with Coordination-Induced Drug Loading for Intracellular Delivery. J. Mater. Sci. Technol..

[76] Rezaei M., Abbasi A., Dinarvand R., Jeddi-Tehrani M., Janczak J. (2018). Design and Synthesis of a Biocompatible 1D Coordination Polymer as Anti-Breast Cancer Drug Carrier, 5-Fu: In Vitro and in Vivo Studies. ACS Appl Mater. Interfaces.

[77] Alvarez E., Marquez A.G., Devic T., Steunou N., Serre C., Bonhomme C., Gervais C., Izquierdo-Barba I., Vallet-Regi M., Laurencin D. (2013). A Biocompatible Calcium Bisphosphonate Coordination Polymer: Towards a Metal-Linker Synergistic Therapeutic Effect?. CrystEngComm.

[78] Wu Y., Zhang F., Wang K., Luo P., Wei Y., Liu S. (2019). Activatable Fluorescence Imaging and Targeted Drug Delivery via Extracellular Vesicle-Like Porous Coordination Polymer Nanoparticles. Anal. Chem..

[79] Gao P.F., Zheng L.L., Liang L.J., Yang X.X., Li Y.F., Huang C.Z. (2013). A New Type of pH-Responsive Coordination Polymer Sphere as a Vehicle for Targeted Anticancer Drug Delivery and Sustained Release. J. Mater. Chem. B.

[80] Huxford R.C., Dekrafft K.E., Boyle W.S., Liu D., Lin W. (2012). Lipid-Coated Nanoscale Coordination Polymers for Targeted Delivery of Antifolates to Cancer Cells. Chem. Sci..

[81] He C., Liu D., Lin W. (2015). Self-Assembled Core-Shell Nanoparticles for Combined Chemotherapy and Photodynamic Therapy of Resistant Head and Neck Cancers. ACS Nano.

[82] He C., Duan X., Guo N., Chan C., Poon C., Weichselbaum R.R., Lin W. (2016). Core-Shell Nanoscale Coordination Polymers Combine Chemotherapy and Photodynamic Therapy to Potentiate Checkpoint Blockade Cancer Immunotherapy. Nat. Commun..

[83] Zhang D., Wu M., Cai Z., Liao N., Ke K., Liu H., Li M., Liu G., Yang H., Liu X. (2018). Chemotherapeutic Drug Based Metal–Organic Particles for Microvesicle-Mediated Deep Penetration and Programmable pH/NIR/Hypoxia Activated Cancer Photochemotherapy. Adv. Sci..

[84] Lu K., He C., Lin W. (2015). A Chlorin-Based Nanoscale Metal-Organic Framework for Photodynamic Therapy of Colon Cancers. J. Am. Chem. Soc..

[85] Guan Q., Li Y.A., Li W.Y., Dong Y.B. (2018). Photodynamic Therapy Based on Nanoscale Metal–Organic Frameworks: From Material Design to Cancer Nanotherapeutics. Chem. Asian J..

[86] Agostinis P., Berg K., Cengel K.A., Foster T.H., Girotti A.W., Gollnick S.O., Hahn S.M., Hamblin M.R., Juzeniene A., Kessel D. (2017). Photodynamic Therapy of Cancer: An Update. CA. Cancer J. Clin..

[87] Ash C., Dubec M., Donne K., Bashford T. (2017). Effect of wavelength and beam width on penetration in light-tissue interaction using computational methods Lasers. Med. Sci..

[88] Sandell J.L., Zhu T.C. (2011). A review of in-vivo optical properties of human tissues and its impact on PDT. J. Biophotonics..

[89] Mallidi S., Anbil S., Bulin A.L., Obaid G., Ichikawa M., Hasan T. (2016). Beyond the Barriers of Light Penetration: Strategies, Perspectives and Possibilities for Photodynamic Therapy. Theranostics.

[90] Huang Z., Huang L., Huang Y., He Y., Sun X., Fu X., Xu X., Wei G., Chen D., Zhao C. (2017). Phthalocyanine-Based Coordination Polymer Nanoparticles for Enhanced Photodynamic Therapy. Nanoscale.

[91] Yang Y., Zhu W., Feng L., Chao Y., Yi X., Dong Z., Yang K., Tan W., Liu Z., Chen M. (2018). G-Quadruplex-Based Nanoscale Coordination Polymers to Modulate Tumor Hypoxia and Achieve Nuclear-Targeted Drug Delivery for Enhanced Photodynamic Therapy. Nano Lett..

[92] Liu J., Tian L., Zhang R., Dong Z., Wang H., Liu Z. (2018). Collagenase-Encapsulated pH-Responsive Nanoscale Coordination Polymers for Tumor Microenvironment Modulation and Enhanced Photodynamic Nanomedicine. ACS Appl. Mater. Interfaces.

[93] Calabrese C.M., Merkel T.J., Briley W.E., Randeria P.S., Narayan S.P., Rouge J.L., Walker D.A., Scott A.W., Mirkin C.A. (2015). Biocompatible Infinite-Coordination-Polymer Nanoparticle-Nucleic-Acid Conjugates for Antisense Gene Regulation. Angew. Chem. Int. Ed..

[94] Chao Y., Liang C., Yang Y., Wang G., Maiti D., Tian L., Wang F., Pan W., Wu S., Yang K. (2018). Highly Effective Radioisotope Cancer Therapy With a Non-Therapeutic Isotope Delivered and Sensitized by Nanoscale Coordination Polymers. ACS Nano.

[95] Wang X., Liang J., Zhang C., Ma G., Wang C., Kong D. (2019). Coordination Microparticle Vaccines Engineered from Tumor Cell Templates. Chem. Commun..

[96] Yang Q., Zhou Z., Cui L., Yang H., Yan C., Zhou X., Yang S., Pan L., Zhang X. (2017). Coordination Polymer Hybridized Au Nanocages: A Nanoplatform for Dual-Modality Imaging Guided near-Infrared Driven Photothermal Therapy in Vivo. J. Mater. Chem. B.

[97] Li M., Wang C., Di Z., Li H., Zhang J., Xue W., Zhao M., Zhang K., Zhao Y., Li L. (2019). Engineering Multifunctional DNA Hybrid Nanospheres through Coordination-Driven Self-Assembly. Angew. Chem. Int. Ed..

[98] Wei Y., Xia H., Zhang F., Wang K., Luo P., Wu Y., Liu S. (2019). Theranostic Nanoprobe Mediated Simultaneous Monitoring and Inhibition of P-Glycoprotein Potentiating Multidrug-Resistant Cancer Therapy. Anal. Chem..

[99] Liu S., Pan J., Liu J., Ma Y., Qiu F., Mei L., Zeng X., Pan G. (2018). Dynamically PEGylated and Borate-Coordination-Polymer-Coated Polydopamine Nanoparticles for Synergetic Tumor-Targeted, Chemo-Photothermal Combination Therapy. Small.

